# Preclinical evidence for the use of anti‐Trop‐2 antibody‐drug conjugate Sacituzumab govitecan in cerebral metastasized castration‐resistant prostate cancer

**DOI:** 10.1002/cam4.7320

**Published:** 2024-06-19

**Authors:** Richard Weiten, Max Niemann, Eduard Below, Lea L. Friker, Damian J. Ralser, Marieta Toma, Glen Kristiansen, Oliver Hahn, Sabrina Zechel, Viktor Grünwald, Tobias Bald, Johannes Siewert, Torsten Pietsch, Manuel Ritter, Michael Hölzel, Markus Eckstein, Abdullah Alajati, Philipp Krausewitz, Niklas Klümper

**Affiliations:** ^1^ Department of Urology and Paediatric Urology University Hospital Bonn Bonn Germany; ^2^ Department of Urology Uro‐Oncology, Robot‐Assisted and Specialized Urologic Surgery University Hospital Cologne Köln Germany; ^3^ Institute of Experimental Oncology University Hospital Bonn Bonn Germany; ^4^ Institute of Neuropathology University Hospital Bonn Bonn Germany; ^5^ Department of Gynaecology and Gynaecological Oncology University Hospital Bonn Bonn Germany; ^6^ Institute of Pathology University Hospital Bonn Bonn Germany; ^7^ Department of Urology University Hospital Göttingen Göttingen Germany; ^8^ Institute of Neuropathology University Hospital Göttingen Göttingen Germany; ^9^ Clinic for Internal Medicine (Tumor Research) and Clinic for Urology, Interdisciplinary Genitourinary Oncology at the West‐German Cancer Center University Hospital Essen Essen Germany; ^10^ Institute of Pathology University Hospital Erlangen, Friedrich‐Alexander‐Universität Erlangen‐Nürnberg Erlangen Germany

**Keywords:** antibody‐drug conjugates, cerebral metastasized CRPC, prostate cancer, Sacituzumab govitecan, Trop‐2

## Abstract

**Purpose:**

Improved survival rates have been observed in castration‐resistant prostate cancer (CRPC) due to advancements in treatment options. However, individuals with brain metastases still have limited therapeutic options and an unfavorable prognosis. Therefore, there is an urgent need to explore new therapeutic avenues, such as antibody‐drug conjugates (ADCs), which have demonstrated significant clinical activity against active brain metastases in solid tumors. Our objective was to determine the expression levels of the ADC targets Trop‐2 and NECTIN‐4 in cerebral metastasized CRPC (mCRPC).

**Methods:**

Immunohistochemical staining of Trop‐2 and NECTIN‐4 with evaluation of H‐score was performed in CRPC brain metastases (*n* = 31). Additionally, we examined Trop‐2 protein expression in prostate cancer cell lines and studied their responsiveness to the anti‐Trop‐2 ADC Sacituzumab govitecan (SG) in vitro.

**Results:**

Our analysis revealed that most patients exhibited moderate to strong Trop‐2 expression [*n* = 27/31 with H‐score ≥100, median H‐score 220 (IQR 180–280)], while NECTIN‐4 was absent in all cerebral metastases. Mechanistically, we demonstrated that the efficacy of SG depends on Trop‐2 expression levels in vitro. Overexpression of Trop‐2 in Trop‐2‐negative PC‐3 cells led to sensitization to SG, whereas CRISPR‐Cas9‐mediated knockdown of Trop‐2 in Trop‐2‐expressing DU‐145 cells conferred resistance to SG.

**Conclusion:**

The substantial expression of Trop‐2 in cerebral metastases, along with our preclinical in vitro results, supports the efficacy of SG in treating cerebral mCRPC. Thus, our results extend the understanding of the potential of ADCs in prostate cancer treatment and provide an additional treatment strategy for the challenging subset of patients with cerebral metastases.

## 1 INTRODUCTION

The incidence of brain metastases in patients with castration‐resistant prostate cancer (CRPC) is gradually increasing, primarily attributed to improved systemic therapies. Persisting as a rare event, with an incidence below 1%.^1^, ^2^ However, these patients are confronted with limited therapeutic options and a significantly compromised prognosis.^3^ Despite the utilization of new antiandrogen therapies and chemotherapies, treatment remains challenging in this subset and there is a high clinical need to explore new therapeutic strategies.^4^, ^5^ Additionally, there is limited evidence for the effectiveness of approved drugs in cerebral mCRPC, as these patients are often excluded from contemporary clinical trials.^4^, ^5^

In this context, antibody‐drug conjugates (ADCs) have emerged as a promising new class of drugs with high efficacy in the treatment of cerebral metastasized solid tumors, for example, Her‐2 targeting ADC for cerebral metastasized breast cancer (Phase II TUXEDO trial).^6^

Previous investigations have demonstrated an upregulation of Trophoblast cell surface antigen‐2 (Trop‐2) across various human solid tumors, including both primary and metastasized prostate cancer, which is associated with an increased risk of disease progression and unfavorable clinical outcomes.^7^ Moreover, Trop‐2 has been identified as a potential therapeutic target for mCRPC treatment.^7^, ^8^ In the IMMU‐132‐01 phase I/II trial (NCT01631552) the efficacy of Sacituzumab govitecan (SG), an anti‐Trop‐2 ADC, which is already approved for treating patients with metastatic urothelial carcinoma (mUC) or triple‐negative breast cancer (TNBC),^9^ has been investigated in patients with advanced epithelial cancers, including 11 patients with mCRPC. SG exhibited a clinical benefit rate, defined as an objective response or stable disease ≥6 months, of 27.3% (*n* = 3/11) in CRPC patients.^7^, ^10^

Similar to TROP‐2, NECTIN‐4 overexpression has been reported in various tumor types,^11^, ^12^ and is linked with several facets of tumor progression, like proliferation, recurrence, and metastasis.^11^, ^12^ Hence, alongside Trop‐2, NECTIN‐4 provides a promising therapeutic target across different cancer types. Furthermore, the effectiveness of Enfortumab vedotin, an anti‐NECTIN‐4 ADC, which has already received approval for treating patients with metastatic urothelial cancer,^13^ is presently under assessment in the ENCORE phase II trial (NCT0475191) for mCRPC.

In this study, we aimed to investigate the expression levels of Trop‐2 and NECTIN‐4 in cerebral metastases of patients with CRPC to assess the potential use of ADC in this hard‐to‐treat population.

## 2 MATERIALS AND METHODS

### 2.1 Patient cohort

We conducted a retrospective analysis of two cohorts comprising in total *n* = 31 patients with cerebral metastasized CRPC treated at the University Medical Center Bonn (*n* = 10) and University Medical Center Göttingen (*n* = 21). Clinicopathologic data of the discovery cohort #1 from the University Hospital Bonn (UKB) are summarized in Table S1. For the validation cohort #2 from the University Hospital Göttingen (UMG) no data were available. 60% (*n* = 6/10) of the patients received a novel antiandrogen therapy and 50% (*n* = 5/10) a taxane‐based chemotherapy. Additionally, *n* = 7/10 patients exhibited an additional metastatic site.

Two independent pathologists (M. Toma, and M. Eckstein) evaluated H‐score for membranous NECTIN‐4 and Trop‐2 staining. The study was approved by the ethical review board of the Medical Faculty of the University of Bonn (approval number: 372/21) and conducted in accordance with the Declaration of Helsinki. All patients provided written informed consent.

### 2.2 Immunohistochemistry

The detection of NECTIN‐4 and Trop‐2 protein was performed through immunohistochemical (IHC) staining on a VENTANA BenchMark ULTRA autostainer (Ventana Medical System) in accordance with an accredited staining protocol in a routine IHC laboratory. The primary antibodies used were a monoclonal anti‐NECTIN‐4 antibody (#ab251110, Abcam, dilution of 1:1000, incubated for 32 min at 37°C) and a monoclonal anti‐Trop‐2 antibody (#ENZ‐ABS380‐0100, Enzo, dilution 1:1500, incubated for 32 min at 37°C). The antigen retrieval was performed using Cc1 buffer from Ventana for 64 min at 91°C. Samples were then classified as negative (H‐score 0–14), weak (H‐score 15–99), moderate (H‐score 100–199), and strong (H‐score 200–300), as previously described.^14^, ^15^

### 2.3 Cell lines and culture conditions

Human prostate cancer cell lines 22Rv1 (derived from a xenograft, CWR22R), PC‐3 (representing a bone metastasis), DU‐145 (representing a brain metastasis), LNCaP and C4‐2B (representing a lymph node metastasis), BPH‐1 (representing a benign prostate hyperplasia) and human embryonic kidney cell line HEK 293 (for viral transduction) provided by American Type Culture Collection (ATCC, Manassas, Virginia) were cultured in RPMI 1640 (#31870‐025, ThermoFisher Scientific) or DMEM medium (Life Technologies Gibco) supplemented with 10% heat‐inactivated foetal calf serum, 0,8% streptomycin‐penicillin antibiotics (10.000 units/mL Penicillin and 10.000 μg/mL Streptomycin; #15140‐122, ThermoFisher Scientific) and 1% L‐glutamine (200 mM; #25030‐024, ThermoFisher Scientific). The cell cultures were incubated at 37°C in a humid environment containing 5% carbon dioxide.

### 2.4 Generation of Trop‐2 single‐guide RNA CRISPR‐Cas9 plasmids

pSpCas9(BB)‐2A‐GFP (PX458) was a gift from Feng Zhang (Addgene plasmid #48138; http://n2t.net/addgene:48138; RRID: Addgene_48138). Generation and cloning of Trop‐2 single‐guide RNA (sgRNA) CRISPR‐Cas9 KO plasmids was performed following the established STAR protocol.^16^ PX458, was digested with the restriction enzyme BbsI (#R0539L, New England Biolabs). Two different sgRNA sequences targeting the Trop‐2 gene were cloned into PX458. The following sgRNAs were used for cloning (Trop‐2 targeting sequences in uppercase letters):

1. Trop‐2_KO1_TS: caccgCACGCGCTCGTGGACAACGA.
2. Trop‐2_KO1_BS: AAACtcgttgtccacgagcgcgtgc.
3. Trop‐2_KO2_TS: caccgCCACCATCCAGATCGAGCTG.
4. Trop‐2_KO2_BS: AAACcagctcgatctggatggtggc.

### 2.5 Establishment of polyclonal Trop‐2 KO cultures

DU‐145 cells were transfected with the Trop‐2 KO plasmids using FuGENE Transfection Reagent (#E2311, Promega) with a FuGENE Transfection Reagent to DNA Ratio of 4:1 (14). 72 h after transfection, the 15% strongest GFP‐positive cells were sorted by Flow Cytometry Core Facility (FCCF; https://btc.uni‐bonn.de/fccf/) of the UKB using the FACS Aria III (BD Biosciences) to obtain polyclonal DU‐145 Trop‐2‐ KO cultures.

### 2.6 Retroviral transduction of Trop‐2 expression plasmid

For retroviral transduction of Trop‐2 overexpressing cultures (PC‐3 OV) a pRP‐Trop‐2 vector was utilized. Retroviral particles were produced in HEK 293 cells by transfecting 1 μg of the retroviral pCL‐Eco and 5 μg of pRP‐Trop‐2. The next day, the medium was replaced by fresh medium. On Day 2, the supernatant was harvested, sterile flirted, and applied on the target cells at different concentrations (50, 75, and 100% virus media) followed by selection with puromycin.

### 2.7 Western Blot

Human PCa cell lines (22Rv1, PC‐3, DU‐145, LNCaP, C4‐2B), were plated in six‐well plates and allowed to reach 80%–90% confluency. The cells were then collected and lysed with RIPA lysis buffer containing protease inhibitors. The protein concentration was determined using the BCA protein assay (#23225, Pierce BCA Protein Assay Kit, ThermoFisher Scientific), after this the samples were added with 4x SDS Sample Loading Buffer [Tris‐HCl (0,2 mol/L), DTT (0,4 mol/L), SDS (277 mmol/L), 8.0% (w/v) Bromophenol blue (6 mmol/L), Glycerol (4,3 mol/L)] and denatured at 95°C for 5 min. The denatured samples were separated using a 4% SDS gel and transferred onto a 0.45 μm nitrocellulose membrane (#GE10600002, Amersham Protran Premium Western Blotting Membrane, Merck). The membrane was blocked with 5% non‐fat milk in TBST (50 nM Tris, 150 nM NaCl, 0.05% Tween 20, pH 7.5) for 60 min and then incubated overnight at 4°C with primary antibodies against Trop‐2 (#ENZ‐ABS380‐0100, Enzo, dilution 1:500) and β‐Actin (#A2228, Sigma dilution 1:5000). HRP‐linked secondary antibodies against mouse (#170‐6516, Bio‐Rad) were applied for 1 h in TBST. The fluorescence signal was detected using the ChemiDoc MP Imaging System (Bio‐Rad).

### 2.8 Flow cytometry

The immunostaining procedure was carried out following standard protocols. Single‐cell suspensions of PC‐3 and DU‐145 were stained with the anti‐human Trop‐2 antibody (#130‐115‐098, Miltenyi Biotec, dilution 1:100) and Zombie NIR (#423106, BioLegend, dilution 1:400). Data were acquired using a BD FACSCanto II flow cytometer (BD Biosciences) and analyzed with the FlowJo software (FlowJo v10.8 BD, https://www.flowjo.com). A total of 1 × 10^4^ cells was measured for each sample.

### 2.9 Measurement of cell viability

To measure the cell viability after treatment with the ADC Sacituzumab govitecan (SG) we used a crystal violet assay (0.05% crystal violet staining, 0.1% acetic acid). The assay involves exposing the cells (1 × 10^4^) to different concentrations of SG (0–10 μg/mL) for 48 h, fixing them with 37% formaldehyde, and staining them with crystal violet. The absorbance of the stained cells is then measured using an ultraviolet–visible spectrometer [570 nm, Safire Reader (Tecan)], and the relative viability of the cells is calculated based on the absorbance values.

### 2.10 Analysis of the SG payload, SN‐38 on PCa cell lines

To evaluate the cytotoxic effect of the payload of SG (SN‐38, a topoisomerase‐1 inhibitor) on well‐established PCa cell lines in‐silico analysis of DepMap portal (https://depmap.org/portal/) was used. Analysis was performed for PCa cell lines 22Rv1, PC‐3, LNCaP, and DU‐145. The curves illustrate a dose‐dependent inhibition of SN‐38 in DU‐145 and PC‐3, as depicted in Figure S1.

### 2.11 Statistical analysis

Statistical analysis was performed using SPSS (Version 28.0.1.1) and GraphPad Prism (Version 9.4.0). Comparisons between two groups were statistically tested with a non‐parametric Mann–Whitney test and a parametric *t*‐test. All *p*‐values were calculated two‐sided, and a *p* < 0.05 was considered statistically significant.

## 3 RESULTS

### 3.1 Robust membranous Trop‐2 expression in CRPC brain metastases

**FIGURE 1:**
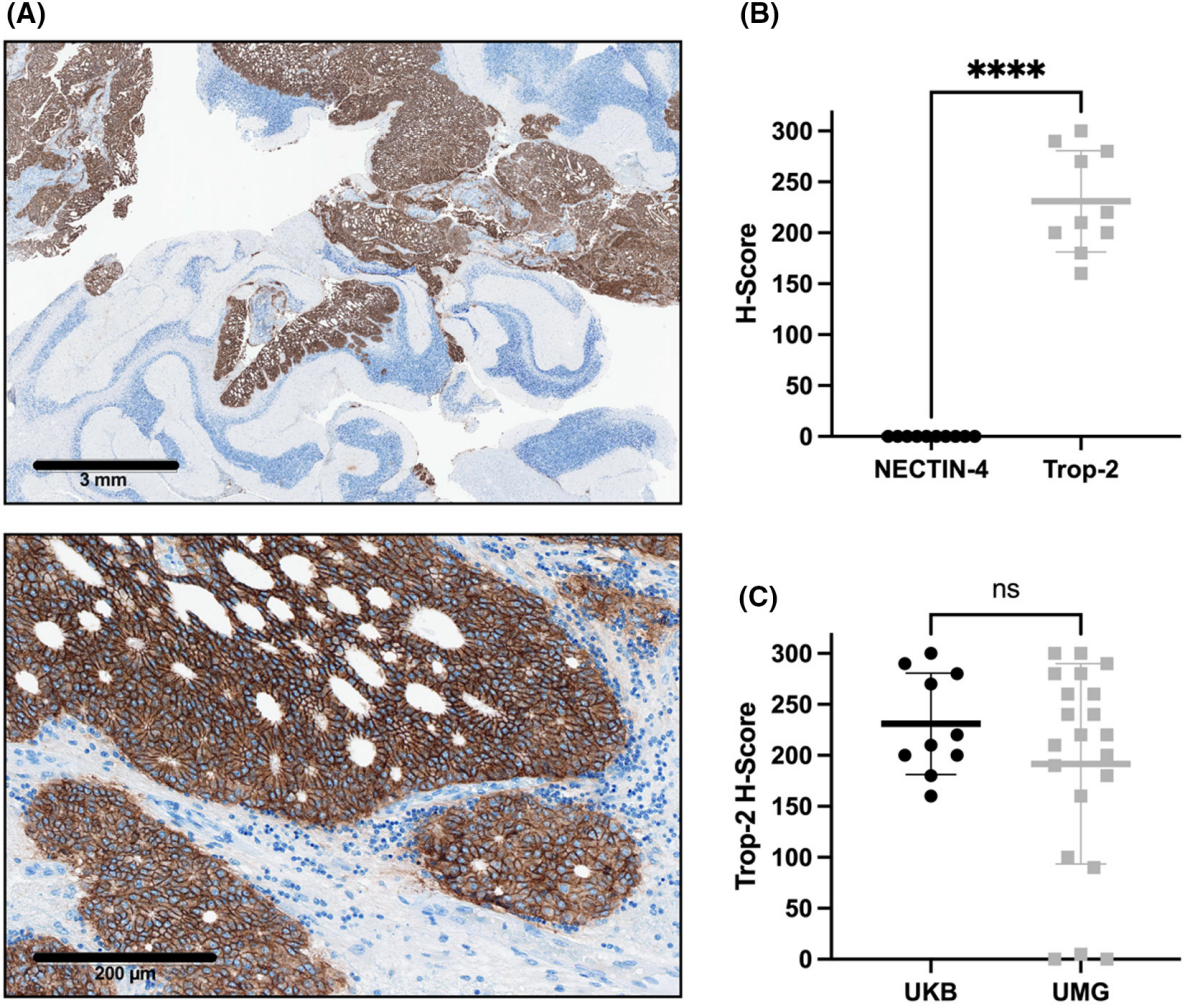
Membranous Trop‐2 protein expression patterns assessed by immunohistochemistry. (A) Representative immunohistochemical staining demonstrates strong membranous Trop‐2 expression (H‐score 290), while adjacent brain tissue is Trop‐2 negative (200× magnification). (B) Moderate to strong membranous Trop‐2 expression in all mCRPC samples of cohort #1 (*n* = 10/10) [median H‐score = 215; interquartile range (IQR) 195–282.5], whereas NECTIN‐4 was absent in all samples (*p* < 0.001). C, Comparable Trop‐2 expression between discovery cohort #1 (UKB) and validation cohort #2 (UMG) (Mann–Whitney test, *p* = 0.5). mCRPC, metastasized castration‐resistant prostate cancer; UKB, University Hospital Bonn, UMG, University Hospital Göttingen. *<0.05, **<0.01, ***<0.001, ****<0.0001.

First, we evaluated the membranous expression of the two ADC targets Trop‐2 and NECTIN‐4 in a discovery cohort consisting of *n* = 10 patients diagnosed with cerebral mCRPC [discovery cohort #1 from the University Hospital Bonn (UKB)]. Subsequently, we validated our findings by analyzing an independent validation cohort comprising *n* = 21 cerebral CRPC specimens [validation cohort #2 from the University Hospital Göttingen (UMG)]. To assess the membranous expression of the ADC targets, we performed immunohistochemical staining with subsequent evaluation of the H‐score.^15^ The representative images of Trop‐2 immunohistochemical staining on CRPC brain metastasis demonstrate robust membranous expression of Trop‐2 in the tumor cells, while the surrounding brain tissue consistently shows no Trop‐2 expression (Figure 1A). In the discovery cohort #1, 100% (*n* = 10/10) of the cerebral mCRPC samples exhibited moderate to strong membranous Trop‐2 expression, defined as H‐score ≥100 as previously described by us and others,^14^, ^15^ with a median H‐score of 215 (IQR 195–282.5), whereas NECTIN‐4 was absent in all samples (Figure 1B). In validation cohort #2, moderate to strong membranous Trop‐2 expression was observed in 80.9% (*n* = 17/21) of cerebral mCRPC samples, with a median H‐score of 220 (IQR 130–270), while only 1 (=4,8%) sample displayed the weak membranous expression of Trop‐2 (H‐score 90). Of note, only three samples (=14.3%) of cohort #2 showed no Trop‐2 expression (Figure 1B). Histopathological analysis revealed that those three tumor samples lacking Trop‐2 expression were all neuroendocrine dedifferentiated. There was no statistically significant difference between cohort #1 and #2 (*p* = 0.5) regarding Trop‐2 expression.

### 3.2 On‐target efficacy of Sacituzumab govitecan in PCa cell lines

**FIGURE 2:**
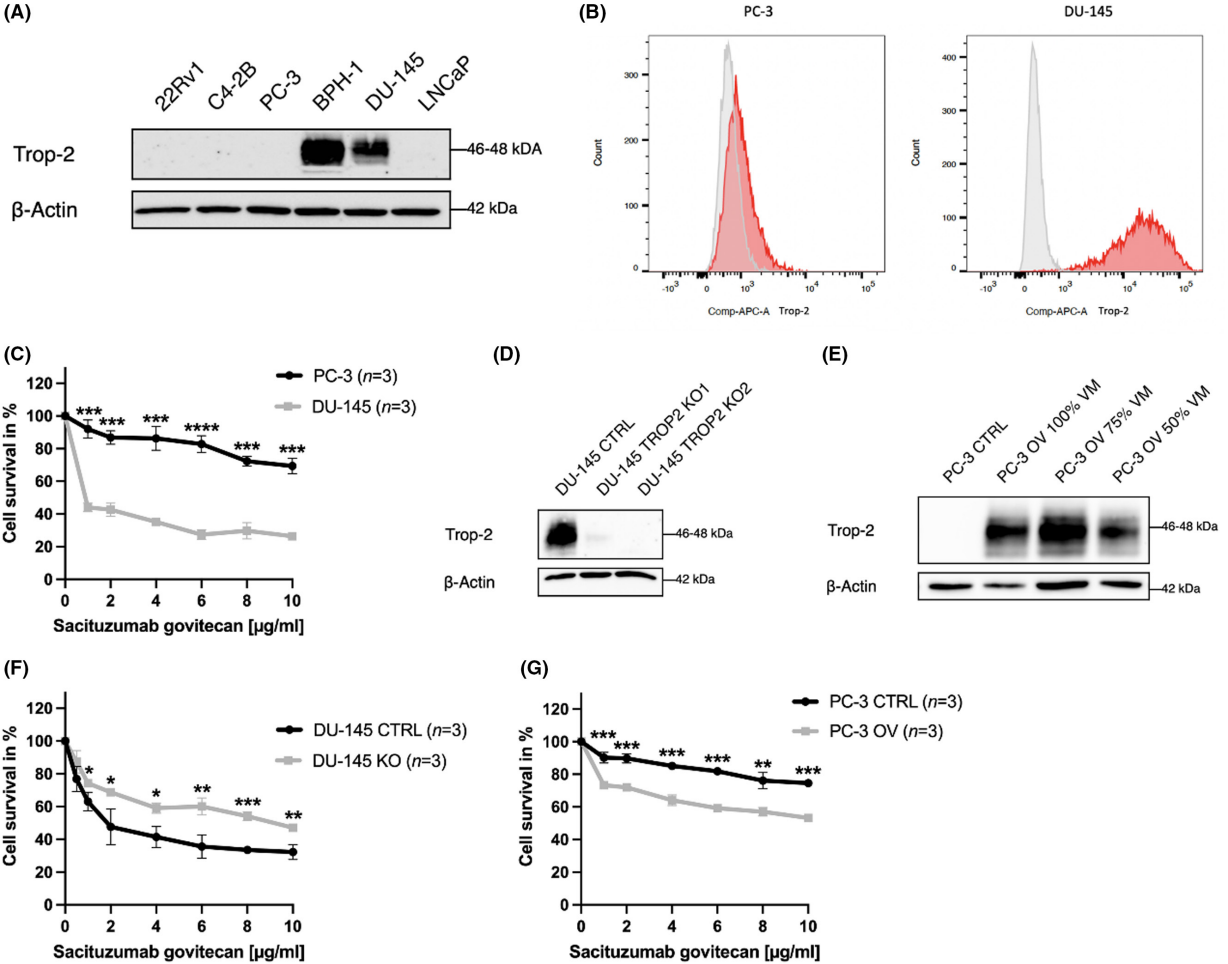
On‐target efficacy of Sacituzumab govitecan in prostate cancer cell lines. (A) Trop‐2 protein expression levels in a panel of PCa cell lines (22Rv1, C4‐2B, PC‐3, DU‐145, and LNCaP) using Western blotting, with only BPH‐1 (non‐malignant prostate cell line) and DU‐145 cells displaying a strong Trop‐2 expression. Detection of b‐Actin served as loading control. (B) Normalized histogram illustrating membranous Trop‐2 expression detected by flow cytometry in DU‐145 WT and PC‐3 (plus unstained control as reference). (C) SG led to significant growth inhibition in the Trop‐2‐expressing DU‐145, while Trop‐2 negative PC‐3 cells were found to be resistant to SG treatment (*t*‐test, *p* < 0.001). D, CRISPR‐Cas9 mediated Trop‐2 knockout (KO) was induced in DU‐145 cells using two sgRNA (KO‐1 and KO‐2) compared with DU‐145 CTRL. Detection of β‐Actin served as loading control. E, Validation of Trop‐2 overexpression in PC‐3 OV cultures by transfecting with three different concentrations of virus media (50%, 75%, and 100%) compared with PC‐3 CTRL. Detection of β‐Actin served as loading control. F, Loss of Trop‐2 (DU‐145 KO) led to SG resistance, G, while overexpression of Trop‐2 led to sensitization to SG in PC‐3. PCa, prostate cancer; SG, Sacituzumab govitecan. *<0.05, **〈0.01, ***<0.001, ****<0.0001.

To assess Trop‐2 expression levels, Western blotting, and flow cytometry were performed on a panel of prostate cancer (PCa) cell lines, including 22Rv1, C4‐2B, PC‐3, DU‐145, and LNCaP. Among these cell lines, only DU‐145 exhibited strong Trop‐2 protein expression (Figure 2A,B). Furthermore, the efficacy of SG on PCa cell growth in vitro was evaluated by treating DU‐145 and PC‐3 cells with varying concentrations of SG. The results showed that SG significantly inhibited cell growth in Trop‐2‐expressing cancer cells (DU‐145), while Trop‐2 negative PC‐3 cells exhibited resistance to SG treatment (*p* < 0.001) (Figure 2C).

To validate the on‐target activity of SG, we established CRISPR‐Cas9‐induced polyclonal Trop‐2 knockout cultures in the Trop‐2 positive DU‐145 cell line using two independent sgRNAs (DU‐145 KO‐1 and KO‐2), and Trop‐2 overexpression (PC‐3 OV) in the Trop‐2 negative PC‐3 cell line via retroviral transduction. Strongly decreased Trop‐2 protein expression was found in both DU‐145 KO‐1 and KO‐2 (Figure 2D). Overexpression of Trop‐2 was induced in PC‐3 OV cultures by transfecting with three different virus media concentrations (50%, 75%, and 100%) compared with PC‐3 cells (Figure 2E). The CRISPR‐Cas9 mediated depletion of Trop‐2 (DU‐145 KO) conferred SG resistance (*p* < 0.001, Figure 2F), as has been previously shown by Chou et al.^8^ Conversely, overexpression of Trop‐2 in SG‐resistant PC‐3 cells led to sensitization to SG (*p* < 0.001, Figure 2G).

## 4 DISCUSSION

In this report, we demonstrated that Trop‐2 is highly expressed in cerebral CRPC metastases, and provided a compelling preclinical rationale for investigating SG treatment for patients with cerebral mCRPC. In addition, the current findings align with previous research that Trop‐2 is overexpressed in CRPC, and targeting Trop‐2 induced dose‐dependent inhibition of cell growth in Trop‐2 expressing cancer cells.^17^

The strong expression of Trop‐2 on tumor cells, coupled with the absence of Trop‐2 expression in adjacent brain tissue and the ability of the SG payload SN‐38 to cross the blood–brain barrier,^18^, ^19^ may lead to a significant therapeutic opportunity. In a subgroup analysis of TNBC patients with brain metastases from the phase 3 ASCENT trial, SG demonstrated a clinical benefit without regarding the intracranial efficacy of SG in breast cancer (BC) brain metastases.^20^ Based on preclinical evidence showing intracranial accumulation of SG and survival benefit in murine xenograft models of BC,^21^ the central nervous system (CNS) activity of SG is being evaluated in a trial involving patients with BC brain metastases or glioblastoma.^22^ Initial findings revealed therapeutic levels of the payload SN‐38 in brain tumor samples, indicating potential accumulation of SG, and intracranial responses were reported in the first patients.^22^ These findings suggest a relevant therapeutic window for treatment in patients with cerebral metastases of CRPC with no or low on‐target toxicity on adjacent brain tissue.^23^ However, it is crucial to determine whether Trop‐2 expression can serve as a predictive biomarker for predicting the therapeutic response to SG. In addition, clinical studies are necessary to thoroughly evaluate the effectiveness and safety of SG in treating cerebral mCRPC. Careful patient selection is necessary before administering anti‐Trop‐2 ADCs, although the expression of Trop‐2 exceeds 80% in cerebral metastases. To address this issue, complementary biomarkers, for example, the analysis of circulating tumor cells (CTCs) or molecular imaging may be a promising approach for assessing ADC targets in cases of cerebral metastasis.^7^, ^24^ However, further investigations are needed to establish the correlation between the Trop‐2 status in (cerebral) metastases and CTCs. Valuable insights on this matter are expected from an ongoing phase II clinical trial involving SG (NCT03725761), which aims to explore the correlation between CTC status and tissue‐based status in patients with mCRPC. In addition, we envision the use of Trop‐2 PET/CT imaging to non‐invasively capture the expression of Trop‐2 in tumors before initiating Trop‐2‐directed therapy.^25^ Molecular imaging holds great potential for providing valuable information on the ADC target expression status, as has been shown for NECTIN‐4 in urothelial cancer, allowing for more targeted and personalized treatment decisions in the sense of ADC precision oncology.^26^, ^27^

Furthermore, diverse observations have been noted regarding Trop‐2 expression in neuroendocrine prostate cancer, making it an interesting avenue for future research.^7^, ^17^ Presently, conclusive inferences regarding intrinsic resistance to Trop‐2‐targeted ADCs are not feasible due to the limited number of cases. Further investigation involving larger cohorts of these rare subtypes is necessary.

Limitations of our study include the complexity of translating in vitro findings into clinical practice, particularly with regard to ADC payload stability, as spontaneous deconjugation at higher concentrations may also lead to non‐target‐specific cytotoxicity in vitro, and retrospective data collection, which may lead to potential bias, underscoring the need for cautious generalization of results to broader populations. In addition, the sample size is only *n* = 31, but for the specific subset of CRPC with cerebral metastases, this cohort size might be considered appropriate given the rarity of such cases.

In conclusion, the strong expression of Trop‐2 in cerebral mCRPC highlights the potential of SG as a promising therapeutic option for the hard‐to‐treat population of cerebral mCRPC.

## AUTHOR CONTRIBUTIONS

**Richard Weiten:** Conceptualization (equal); data curation (equal); formal analysis (equal); investigation (equal); methodology (equal); project administration (equal); resources (equal); software (equal); visualization (equal); writing – original draft (equal). **Max Niemann:** Conceptualization (equal); formal analysis (equal); investigation (equal); writing – original draft (equal). **Eduard Below:** Methodology (supporting); resources (supporting); writing – review and editing (supporting). **Lea L. Friker:** Data curation (equal); resources (equal); writing – review and editing (equal). **Damian J. Ralser:** Writing – review and editing (equal). **Marieta Toma:** Resources (supporting); writing – review and editing (supporting). **Glen Kristiansen:** Resources (supporting); writing – review and editing (supporting). **Oliver Hahn:** Resources (equal); writing – review and editing (equal). **Sabrina Zechel:** Resources (equal); writing – review and editing (equal). **Viktor Grünwald:** Writing – review and editing (equal). **Tobias Bald:** Methodology (supporting); resources (supporting); writing – review and editing (equal). **Johannes Siewert:** Methodology (supporting); resources (supporting); writing – review and editing (equal). **Torsten Pietsch:** Resources (supporting); writing – review and editing (supporting). **Manuel Ritter:** Writing – review and editing (equal). **Michael Hölzel:** Writing – review and editing (equal). **Markus Eckstein:** Formal analysis (equal); writing – review and editing (equal). **Abdullah Alajati:** Writing – review and editing (supporting). **Philipp Krausewitz:** Conceptualization (equal); data curation (equal); formal analysis (equal); investigation (equal); methodology (equal); project administration (equal); resources (equal); supervision (equal); visualization (equal); writing – original draft (equal). **Niklas Klümper:** Conceptualization (equal); data curation (equal); formal analysis (equal); investigation (equal); project administration (equal); resources (equal); supervision (equal); visualization (equal); writing – original draft (equal).

## FUNDING INFORMATION

This work was supported by the Open Access Publication Fund of the University of Bonn.

## CONFLICT OF INTEREST STATEMENT

DJR: Personal fees, travel costs, and speaker's honoraria from Novartis. OH: Research funding from Janssen‐Cilag; advisory role, travel costs, personal fees, and speaker fees from Astellas, AstraZeneca, Bayer, BMS, Eisai, and Medac. SZ: Speaker's honoraria from Sanofi. VG: Research funding from AstraZeneca, Novartis, BMS, MSD, Ipsen, Pfizer; honoraria and consultation fees from AstraZeneca, BMS, Novartis, Amgen, Astellas, Apogepha, Ipsen, EISAI, MSD, MerckSerono, Roche, EUSAPharm, Janssen, ONO Pharmaceutical, cureteq, Debiopharm, PCI Biotech, Oncorena, Novartis/AAA, Gilead; stocks shareholder from AstraZeneca, BMS, SeaGen, MSD, GenMab; travel expenses AstraZeneca, BMS, MerckSerono; Janssen. MR: Speaker's honoraria from medac; advisory role for Siemens Healthineers; research funding from Procept BioRobotics. MH: Research funding by TME Pharma (Noxxon), honoraria from BMS, Novartis. ME: Personal fees, travel costs and speaker's honoraria from MSD, AstraZeneca, Janssen‐Cilag, Cepheid, Roche, Astellas, Diaceutics; research funding from AstraZeneca, Janssen‐Cilag, STRATIFYER, Cepheid, Roche, Gilead; advisory roles for Diaceutics, MSD, AstraZeneca, Janssen‐Cilag, GenomicHealth. PK: Personal fees, travel costs, and speaker's honoraria from Bayer, Janssen‐Cilag, Medac. NK: Personal fees, travel costs, and speaker's honoraria from Astellas, Novartis, Ipsen, Photocure, MSD. All other authors declare no conflict of interest.

## ETHICS STATEMENT

The study was approved by the ethical review board of the Medical Faculty of the University of Bonn (approval number: 372/21) and conducted in accordance with the Declaration of Helsinki.

## CONSENT

All patients provided written informed consent.

## Supporting information


Data S1:

## Data Availability

The data that support the findings of this study are available from the corresponding author upon reasonable request.
