# Supplementary material for: Preclinical evidence for the use of anti‐Trop‐2 antibody‐drug conjugate Sacituzumab govitecan in cerebral metastasized castration‐resistant prostate cancer
Source: Cancer Med. 2024 Jun 19;13(12):e7320. doi: 10.1002/cam4.7320 (PMC11185941; doi:10.1002/cam4.7320)
Supplement: Supplementary file 1 — Data S1: [file CAM4-13-e7320-s001.docx]

**Supporting Information(s)**

**Table S1.** Clinicopathological characteristics for the discovery #1 cohort from the University Hospital Bonn (UKB) (*n*=10).

**Figure S1.** Dose-dependent inhibition of SN-38, a topoisomerase-1 inhibitor, on PCa cell lines, 22Rv1 (A), PC-3 (B), DU-145 (C), and LNCaP (D) using DepMap portal (https://depmap.org/portal/).
